# Fragmentation of magnetism in artificial kagome dipolar spin ice

**DOI:** 10.1038/ncomms11446

**Published:** 2016-05-13

**Authors:** Benjamin Canals, Ioan-Augustin Chioar, Van-Dai Nguyen, Michel Hehn, Daniel Lacour, François Montaigne, Andrea Locatelli, Tevfik Onur Menteş, Benito Santos Burgos, Nicolas Rougemaille

**Affiliations:** 1 CNRS, Inst NEEL, F-38000 Grenoble, France; 2 Univ. Grenoble Alpes, Inst NEEL, F-38000 Grenoble, France; 3 Institut Jean Lamour, Université de Lorraine and CNRS, Vandoeuvre lès Nancy, F-54506, France; 4 Elettra-Sincrotrone Trieste S.C.p.A., SS 14, km 163.5, AREA Science Park, Basovizza, 34149 Trieste, Italy

## Abstract

Geometrical frustration in magnetic materials often gives rise to exotic, low-temperature states of matter, such as the ones observed in spin ices. Here we report the imaging of the magnetic states of a thermally active artificial magnetic ice that reveal the fingerprints of a spin fragmentation process. This fragmentation corresponds to a splitting of the magnetic degree of freedom into two channels and is evidenced in both real and reciprocal space. Furthermore, the internal organization of both channels is interpreted within the framework of a hybrid spin–charge model that directly emerges from the parent spin model of the kagome dipolar spin ice. Our experimental and theoretical results provide insights into the physics of frustrated magnets and deepen our understanding of emergent fields through the use of tailor-made magnetism.

## Abstract (web summary)

By nanofabricating arrays of dipolar-coupled bistable single-domain nanomagnets, artificial model systems exhibiting collective ordering may be realized. Here, the authors present signatures of spin fragmentation in low-energy states of an artificial kagome ice.

## Graphical abstract

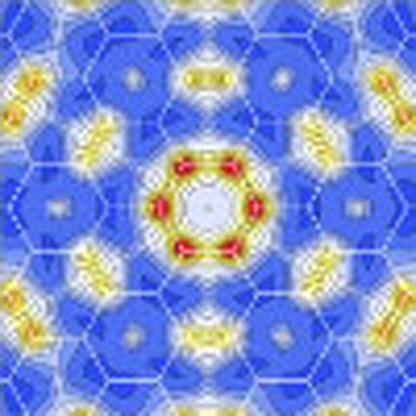

Frustration refers to the inability of a complex system to satisfy all its constraints simultaneously^1^. Geometrical frustration arises when these constraints are driven by the topology of an underlying lattice^2^. Examples of such systems can be found in a wide class of magnetic materials^3^ and artificial magnetic architectures^4^,^5^,^6^,^7^ known as geometrically frustrated magnets. In some cases, these systems are characterized by highly fluctuating ground-state manifolds, often referred to as spin liquids, and can exhibit unusual magnetic excitations such as fractional quasi-particles^8^.

The frustrated kagome Ising antiferromagnet is one of the first studied classical spin liquids, that is, a strongly correlated magnetic model exhibiting only short-range spin–spin correlations down to the lowest temperatures^9^,^10^. This short-range antiferromagnetic model can be mapped, one to one, onto a short-range ferromagnetic model, where geometrical frustration is provided by multiaxial Ising-like anisotropies^11^. The thermodynamics of both models is described by two temperature regimes: a high-temperature paramagnet and a low-temperature cooperative paramagnet, in which every triangular unit cell of the kagome lattice obeys the so-called (kagome) ice rule, meaning that two spins are pointing in or out of each triangle of the kagome lattice. This ice-like constraint on a triangle is akin to the original vertex water–ice rule^12^ (hence their name, spin ices), which lies at the heart of the low-energy properties of square^13^ and pyrochlore^14^ spin ices.

Adding long-range dipolar interactions to the short-range frustrated multiaxial ferromagnet drastically modifies its low-temperature behaviour^15^. The first spin-ice manifold (SI1) survives over a finite temperature range and is then followed, at lower temperatures, by a second spin-ice manifold (SI2), in which spin-loop fluctuations coexist with an effective magnetic charge crystal. Eventually, a Néel-like ordering (long-range order—LRO) occurs at the lowest temperatures. In both the SI1 and SI2 phases, the kagome ice rule is obeyed. However, the magnetic charges, associated with the fractionalization of each spin into opposite pole pairs, are in a paramagnetic state in the SI1 phase, while they crystallize in the SI2 phase^15^,^16^. Although more constrained, the SI2 phase remains macroscopically degenerated, as shown by its finite entropy.

The puzzling aspect of the SI2 phase is the emergence of the magnetic charge crystallization, which is *a priori* not encoded in the underlying dipolar spin-ice (DSI) model^15^,^16^. Experimental evidences of charge crystallites in artificial kagome spin ices have been also reported^17^,^18^,^19^,^20^,^21^,^22^ and interpreted with the use of a phenomenological spin–charge model^21^. Here we provide a theoretical framework that allows us to reveal the microscopic origin of this emerging charge organization. Besides, in the particular case of the kagome DSI, the formation of an antiferromagnetic charge crystal is a direct consequence of the recent proposal of spin fragmentation^23^. Furthermore, using thermally active kagome arrays of nanomagnets^18^,^19^,^20^,^21^,^22^,^24^,^25^, we evidence experimental signatures of this fragmentation of magnetism.

## Results

### *Ab initio* coding of emergence

**Figure 1:**
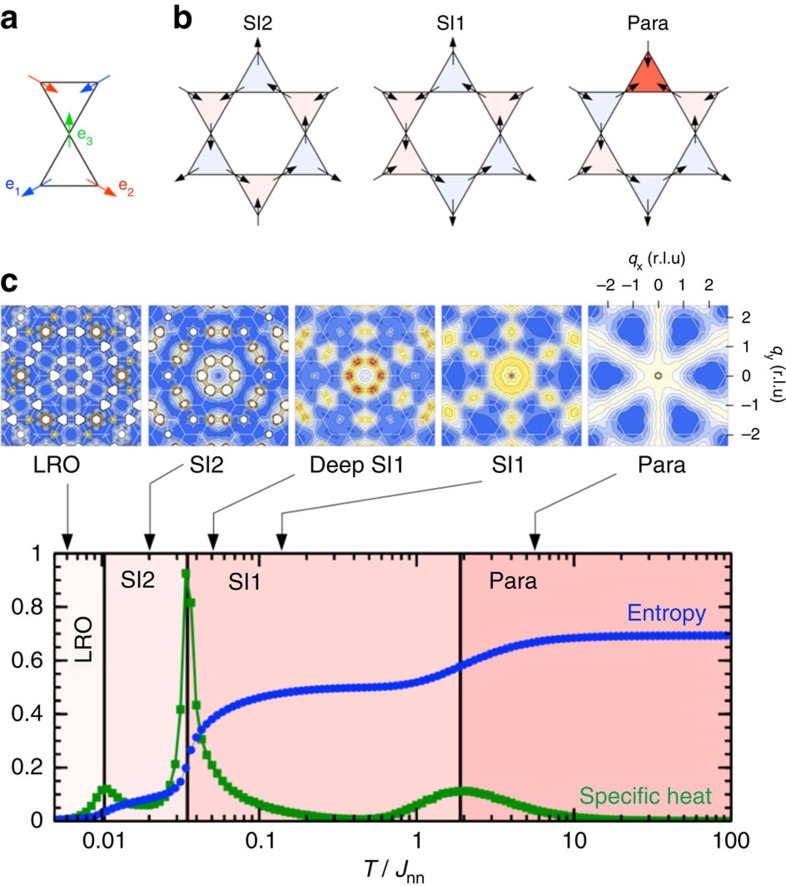
Thermodynamics of the kagome dipolar spin ice. (**a**) The three possible anisotropy directions in the kagome lattice are denoted by the **e**_*i*_ unit vectors, which allow the description of the Ising-like spins as *σ*_*i*_ scalar variables. (**b**) Three examples of magnetic configurations belonging to the spin ice 2 (SI2), spin ice 1 (SI1) and paramagnetic regimes. Each spin at the vertex of the kagome lattice can be seen as a magnetic dipole and fractionalized into an opposite pole pair. By conventionally taking the head of the arrow to be a +1 magnetic charge and the tail as a −1 magnetic charge, a total magnetic charge can be attributed to each triangle, that is, to each vertex of the kagome lattice, by summing the three elementary charges of the fractionalized spins. A total *Q*=+1/−1 is depicted by a light red/blue triangle, respectively. The paramagnetic phase also features ±3 charges, as indicated for instance by a dark-red triangle. As soon as the system enters its spin-ice manifolds, ice rules are unanimously obeyed, which translates into the presence of only unitary charges. While these charges are disordered within the SI1 phase, they eventually crystallize antiferromagnetically in the SI2 phase, as depicted by the alternation of red–blue triangles. (**c**) The simulated temperature dependencies of the entropy and the specific heat of the kagome dipolar spin-ice model. Magnetic structure factor [*S*(**q**)] maps associated to each phase are provided and their corresponding normalized temperatures *T*/*J*_nn_ are 5.815 (paramagnetic), 0.131 (SI1), 0.051 (deep SI1), 0.020 (SI2) and 0.006 (LRO), where *J*_nn_ is the effective nearest-neighbour interaction.

In this section, we discuss how the magnetic charge at the vertices is encoded into the Hamiltonian of the dipolar kagome spin-ice model and we reveal why these charges crystallize at low temperature. To do so, we consider the dipolar Hamiltonian


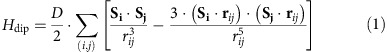


where *D* is the dipolar constant, *r*_*ij*_ the distance between sites *i* and *j*, and **S**_*i*_=*σ*_*i*_**e**_*i*_ an Ising-like spin residing on site *i* and pointing along its local anisotropy axis (Fig. 1a). Once this model is brought to low temperatures, ice rules are unanimously fulfilled or, equivalently, the total magnetic charge at each vertex of the honeycomb lattice is ±1 (Fig. 1b). Since the vertex charges are defined as local sum of the individual magnetic poles of the fractionalized spins, it is this spin/charge correspondence, together with the particular structure of the dipolar interactions in this lattice, which allow the rewriting of the microscopic spin model. The Hamiltonian then becomes a hybrid spin–charge model with only Ising-like variables,


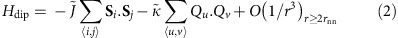


where the first term is a short-range spin-ice kagome ferromagnet (that is, an Ising spin liquid), while the second term corresponds to an antiferromagnet of magnetic charges *Q* on an hexagonal lattice (see the Methods section). The remaining terms correspond to the longer-range part of the dipolar spin model. The sum of these last terms is, algebraically speaking, absolutely convergent and they do not interfere with the physics driven by the first two contributions, except at very low temperatures where they lead to the Néel ordering (LRO).

This exact mapping of equation (1) onto equation (2) explains why charge crystallization is observed in the SI2 phase. As 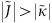, the first term ensures that kagome ice rules are fulfilled when the temperature is lowered, and the model fluctuates within the first spin-ice manifold, SI1. Because the Ising charge–charge interaction is unfrustrated and is fully compatible with the SI1 constraint, it leads to charge crystallization when the temperature is further reduced, that is, it selects a sub-manifold of the SI1 set, the SI2 manifold. We emphasize that this exact algebraic mapping of the original spin model onto a hybrid spin–charge Hamiltonian is one of the very few examples of *ab initio* coding of emergence: the collective organization of magnetic charges is directly related to the original microscopic spin degree of freedom.

### Fragmentation of magnetism in the SI2 phase

Once this model is brought into the SI2 phase, magnetic charges at each vertex are constrained to *Q*=±1. As noted by Brooks-Bartlett *et al*.^23^, a formal analogy with electrostatics can be made and this distribution of unitary magnetic charges can be related to the divergence of the local magnetization, which is proportional to the sum of the spins pointing inwards and outwards of the corresponding triangle of the kagome lattice. This means that


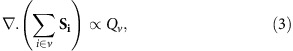


where **S**_**i**_ is one of the three spins participating to the vertex *v* and *Q*_*v*_ the associated unitary charge. Applying a lattice Helmholtz–Hodge decomposition on the vector field {∑_*i*∈*v*_ **S**_**i**_}_*v*_, results in a splitting into a curl-free and a divergence-free contribution,


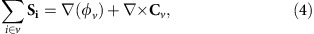


where *ϕ* is a scalar field and 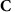 a vector field. From this decomposition, it is clear that only the scalar field *ϕ* carries information on the magnetic charge as


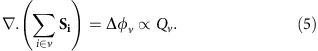


It is worth noting that such a decomposition is simply an algebraic rewriting and can be applied to any vector field defined on a lattice.

The remarkable property of this model is the decoupling of the divergence-free and divergence full channels at low temperatures. Within the SI2 region, the scalar field *ϕ* orders because of the *Q*_*u*_.*Q*_*v*_ term in the Hamiltonian, which leads to the charge crystallization. On the other hand, the first two terms of the hybrid spin–charge Hamiltonian do not impact the vector field 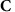, leaving it free to fluctuate. This last property, associated with its natural divergence-free nature, defines a so-called Coulomb phase^26^. This exotic state of matter, within which order and disorder coexist, is very unusual, as both aspects of magnetic organization are carried by the same degree of freedom, hence the name fragmentation.

### Looking for signatures of spin fragmentation

**Figure 2:**
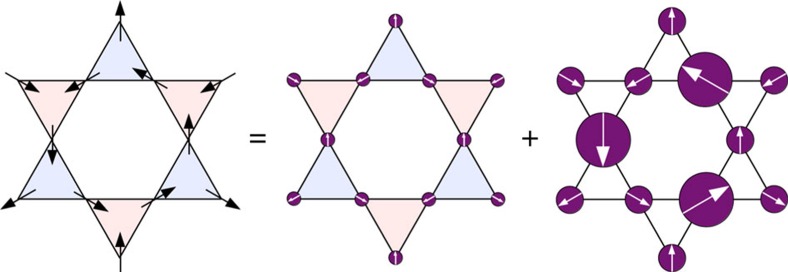
Illustration of the spin fragmentation process. Spins are represented by black arrows, while the associated magnetic charges at the kagome vertices are represented by a red/blue triangle, corresponding to a +1/−1 charge state, respectively. By applying a Helmholtz–Hodge decomposition over the entire network, each spin of the lattice fragments into two channels, such that the magnetic configuration decomposes into a divergence-full and a divergence-free field. Note that, for the two fields, both the spin direction and the spin length change. To better visualize the fragmentation process, fragmented spins are represented by purple circles of diameters 1/3, 2/3 and 4/3, according to the moment of the fragmented part. This type of decomposition can be performed on any spin configuration belonging to the SI2 phase, for which the divergence-full channel is always the same and independent of the initial spin configuration, while the divergence-free channel can fluctuate, ensuring the magnetic equivalent of a Kirchhoff law.

This theoretical framework provides insights into how fingerprints of this fragmentation process can potentially be revealed in experiments. While Fig. 2 details the fragmentation process in real space, a more straightforward way to visualize it is by plotting the magnetic structure factor *S*(**q**), that is, the Fourier transform of spin–spin correlations. In reciprocal space, the static divergence-full channel is revealed through magnetic Bragg peaks, while the divergence-free channel appears as a structured diffuse signal^27^. It is the coexistence of both types of signal which demonstrates spin fragmentation^23^. The temperature dependence of the two-dimensional (2D) magnetic structure factor for the DSI model has been computed using Monte Carlo simulations (see the Methods section) and is reported in Fig. 1c along with the temperature dependence of the entropy and specific heat. In the following analysis of our measurements, it is this coexistence of Bragg peaks and structured diffuse background signal that we use to evidence the spin fragmentation process experimentally. Note that charge crystallization corresponds to a rather counter–intuitive phenomenon. The magnetic charge crystal is the emerging description of an antiferromagnetic all-in/all-out fragmented spin ordering, as depicted in Fig. 2, in a nevertheless ferromagnetic spin model. Real-space imaging of artificial spin-ice systems allows direct visualization of this counter–intuitive phenomenon.

### Experimental evidence of spin fragmentation

**Figure 3:**
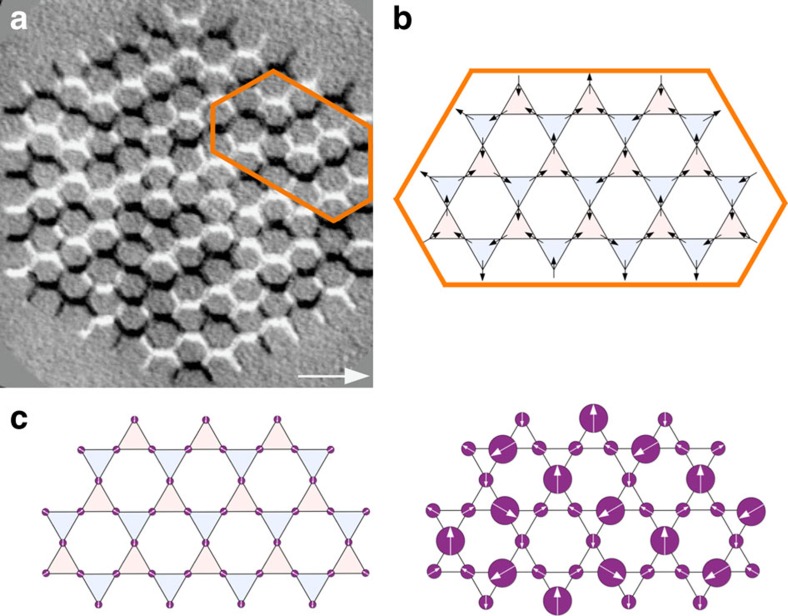
Real space evidence of spin fragmentation. (**a**) XMCD-PEEM (10 × 11 μm^2^) image of an artificial kagome array. The black and white contrasts give the local direction of the magnetization within each individual nanomagnet. The white arrow indicates the direction of the incident X-ray beam. The orange hexagon highlights a region of the array where perfect charge ordering is observed. (**b**) The local spin configuration within the orange hexagon deduced from the XMCD-PEEM image. Spins are represented by black arrows, while the associated magnetic charges at the kagome vertices are represented by a red/blue triangle, corresponding to a +1/−1 charge state, respectively. (**c**) Helmholtz–Hodge decomposition performed on the local spin configuration shown in the orange hexagon. Fragmented spins are represented by purple circles of diameters 1/3, 2/3 and 4/3, according to the moment of the fragmented part.

Evidencing the spin fragmentation process in artificial kagome spin ice is challenging, mainly because of the experimental difficulty to bring such systems into their low-energy manifolds, where collective phenomena emerge. In the following, we show, however, that this can be done in thermally active, kagome arrays of connected Gd_0.3_Co_0.7_ nanomagnets (see the Methods section; Supplementary Note 1; Supplementary Fig. 1), which have been studied using X-ray magnetic circular dichroism-photoemission electron microscopy (XMCD-PEEM) magnetic imaging (see the Methods section). A typical magnetic image is reported in Fig. 3a. Black and white contrasts allow the determination of the magnetization direction within each single nanomagnet. Hence, the overall spin configuration of the array is deduced together with the distribution of the associated magnetic charges at the vertex sites.

**Figure 4:**
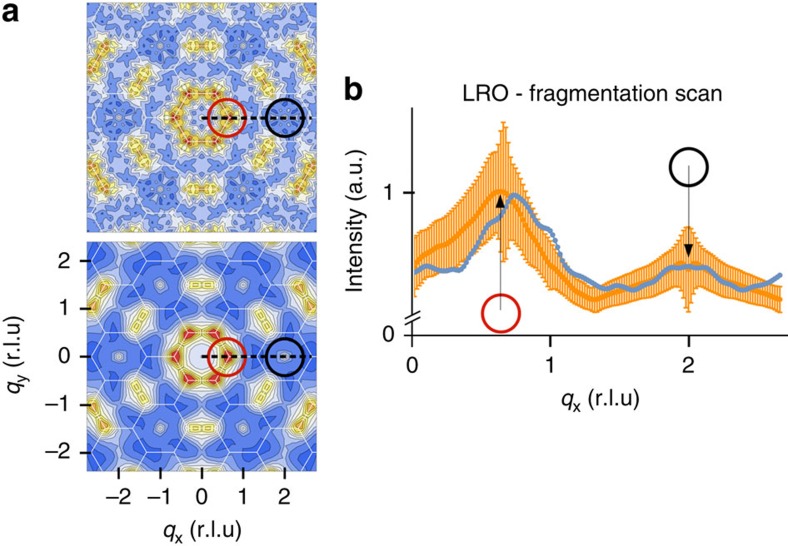
Spin fragmentation in reciprocal space. (**a**) Experimental (top) and theoretical (bottom) 2D maps of the magnetic structure factor corresponding to the experimental XMCD-PEEM image. The positions of the Bragg peaks corresponding to both the fragmented spin phase and to the LRO are indicated by black and red circles, respectively. One particular scan in the reciprocal space is indicated by a dashed black line in the two maps. The scan starts at the origin of the reciprocal space and passes firstly through what will be a LRO Bragg peak, and then through a fragmentation peak. (**b**) Comparison between the experimental (blue) and theoretical (orange) q-scans along the direction mentioned before. S.d. of the theoretical fluctuations (orange) are reported to quantify the likelihood of the dipolar spin-ice model to describe the experimental observations. The dipolar spin-ice model captures most features of spin–spin correlations and agrees semi-quantitatively on the amplitude, as well as on the positions, of the correlations. Intensity is given in arbitrary units, but it must be noted that both experimental and theoretical curves have been scaled in a similar way, that is, there is no free parameter but the effective temperature (*T*/*J*_nn_=0.051) of the Monte Carlo simulations.

Using the spin configuration of the whole lattice obtained from our XMCD-PEEM measurements, we can compute the magnetic structure factor and compare it with the one predicted by Monte Carlo simulations at a similar effective temperature (the effective temperature of our array is estimated by comparing the measured spin–spin correlation coefficients to their thermodynamic values^17^,^18^,^28^, see the Methods section). The result is reported in Fig. 4. At first sight, there is a fairly good qualitative agreement between the experimental (Fig. 4a, top) and theoretical (Fig. 4a, bottom) 2D maps of the magnetic structure factor. The most striking feature of the experimental map consists in the clear fingerprints of a spin fragmentation process that are twofold: appearance of Bragg peaks (see black circles) and a structured diffuse background signal accounting for the disordered phase of the divergence-free component (see yellow regions in the 2D maps). We emphasize again that it is this coexistence that demonstrates spin fragmentation. The sole presence of charge ordering is not sufficient to evidence spin fragmentation, as the key aspect of this phenomenon is the emergent decoupling of the divergence-free and divergence-full spin channels. For example, the LRO ground state or a saturated magnetic configuration would directly relate charge crystallization to spin ordering (that is, there is no fragmentation of magnetism through the decoupling of the two channels).

This qualitative agreement between the experimental and theoretical maps can be made more quantitative. Figure 4b provides a comparison between the experimental and the theoretical magnetic structure factors along a q-scan through the reciprocal space, passing through the fragmentation peak (black circle in Fig. 4a). Because the experimental statistics is low, we have reported the theoretical Monte Carlo signal along the q-scan with its s.d. to quantify the likelihood of the DSI model to describe our measurements. It appears that the DSI model captures quantitatively the fragmentation process. The experimental image also displays several regions of alternating +1 and −1 magnetic charges, pointing to incipient charge-ordered crystallites. This sample has therefore not reached the thermodynamic SI2 phase, within which a unique crystallite would be expected, but is consistent with the effective temperature *T*/*J*_nn_=0.051 deduced for the spin–spin correlator analysis, that places the array deep into the SI1 phase. Thanks to the real-space imaging of this artificial magnet, the lattice Helmholtz–Hodge decomposition can be performed for each crystallite. One of these crystallites is highlighted by an orange hexagon and better illustrated in Fig. 3b. Along with the black arrows that indicate the local spin directions, we use again the red/blue colour code for each kagome triangle to represent the vertex charge state. In this selected region, there is no spin order, although the magnetic charge has crystallized^18^,^19^,^20^,^21^ and is embodied in a fragmented all-in/all-out antiferromagnetic spin ordering (see Fig. 3c). The array has thus been brought, locally, into the SI2 phase.

Finally, we emphasize that our observation of a spin fragmentation process is not limited to the case of our thermally active GdCo alloy spin-ice system. Very similar results have been obtained with more conventional, athermal, permalloy-based kagome arrays that we demagnetized using a field protocol before their imaging in a magnetic force microscope. In such samples, we also observed the coexistence of Bragg peaks and a structured diffuse background signal in the 2D maps of the magnetic structure factor (Supplementary Note 2; Supplementary Table 1; Supplementary Fig. 2), thus proving the generality of the concept and the capability to measure it experimentally using different materials and demagnetization protocols.

## Discussion

In conclusion, we report the experimental signatures of a magnetic-moment fragmentation process in a thermally active artificial magnet and provide a theoretical framework to interpret this emerging phenomenon. We emphasize that the long-range dipolar nature of the interactions in artificial magnetic ices is at the heart of both our experimental and theoretical results. Achieving a complete tuning of the cooling procedure now becomes an ultimate goal, as it would provide a statistical physics laboratory, paving the way for engineered magnetic structures and their use to understand, realize and control new states of matter, be they artificial or not.

## Methods

### Sample fabrication

Our samples were fabricated on Si substrates from full films grown by ultra-high vacuum sputtering (base pressure in the 10^−9^ mbar range). The final stack has the following composition: Si//Ta (5 nm)/Gd_0.3_Co_0.7_ (10 nm)/Ru (2.6 nm). The 5-nm-thick Ta layer is used as a buffer for the subsequent growth of the magnetic GdCo film, which is finally capped with a 2.6-nm-thick Ru layer. The capping material and its thickness have been optimized to protect the sample from oxidation and against chemical treatments during the lithography process, while still keeping high the magnetic contrast in imaging conditions. The Gd_0.3_Co_0.7_ material is a ferrimagnetic alloy characterized by a Curie temperature (*T*_C_) of ∼475 K, which was adjusted by co-sputtering Co and Gd in d.c. mode to control the alloy composition. Additional information on the magnetic properties of the GdCo alloy is provided in the Supplementary Note 1. The arrays that we fabricated using electron-beam lithography and ion-beam etching are composed of 342 nanomagnets having typical dimensions of 500 × 100 × 10 nm^3^. At room temperature, each nanomagnet has a magnetization pointing along the long axis of the element, due to shape anisotropy, and can thus be considered as an Ising pseudo-spin.

### Thermal annealing protocol

Experimentally, the arrays are first saturated using an external magnetic field to set the initial spin configuration in a well-defined state. The arrays are then heated up above the Curie temperature of the material by passing a current through a W-filament underneath the sample stage. The cooling procedure was performed as follow: when reaching the targeted temperature, the filament current was quickly switched off to avoid the presence of an Oersted field while cooling down the sample through *T*_C_. The typical cooling time is 30 min and is basically limited by the thermal dissipation into the sample holder. After cooling down the sample back to room temperature, the resulting spin configurations of the arrays are imaged using X-xay PEEM combined with XMCD. Measurements were carried out at the nanospectroscopy beamline of the Elettra synchrotron, Trieste, Italy.

### Estimation of an effective temperature

Although in a frozen magnetic state (that is, the magnetic configuration does not evolve anymore once the sample is at room temperature) when being imaged, an effective temperature can be associated to the spin configuration resulting from our thermal treatment. To do so, we proceed similarly to what we did in previous works by employing a standard deviation analysis through the use of a ‘spread-out' function based on the spin–spin correlators^18^,^28^. This ‘spread-out' function is defined as: 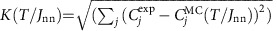, where 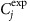 represents the experimental correlations, while 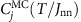 are the average Monte Carlo correlations at a given temperature *T*/*J*_nn_, with *j* ranging from 1, the nearest-neighbour correlation, up to 7 (the 7th next nearest-neighbours). For our set of experimental values, this function can be computed over the entire range of Monte Carlo temperatures. The minimum of *K*(*T*/*J*_nn_) defines the effective temperature. Additional works on artificial spin-ice systems where an effective temperature is associated to an arrested (that is, frozen) microstate can be found for example in refs 29, 30.

### Monte Carlo simulations

The Monte Carlo simulations were performed on 12 × 12 × 3 kagome lattice sites with periodic boundary conditions. This geometry corresponds to the periodic boundary condition cluster having the closest number of sites to the one of the finite experimental realization, while also being compatible with the periodicity of the long-range order expected at the lowest temperatures. 10^4^ modified Monte Carlo steps (MMCSs) are used for thermalization and measurements are computed over 10^4^ MMCSs, where one MMCS involves local spin flips as well as global loop flips, such that the analysis of the integrated correlation time calculated on-the-fly ensures stochastic decorrelation between measurements. We note that simulations performed for a network of 342 spins with free boundary conditions yield very similar results. Furthermore, the same ground-state configuration is found for this particular finite system size as in the infinite network case. Both types of simulations ensure that all the physics at stake, be it experimental or theoretical, does not depend on the cluster geometry or on the boundary conditions.

### Dipolar Hamiltonian

Nanomagnets interact through the magnetostatic interaction. They possess a strong anisotropic shape (aspect ratio of ∼5) and are well approximated by Ising-like variables oriented along their long axis. As shown in ref. 17, the point dipole approximation of the magnetostatic terms is valid only beyond nearest neighbours. For the closest elements, their shape and proximity prevent the dipolar approximation to be valid. It appears however that multipolar terms can actually be taken into account correctly, provided that the nearest-neighbour dipolar term is enhanced by an extra coupling *J*_1_. The full Hamiltonian describing this system then reads


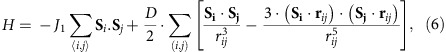


where *D* is the dipolar constant, *r*_*ij*_ the distance between sites *i* and *j*, and **S**_*i*_=*σ*_*i*_**e**_*i*_ the spin residing on the site *i*, with *σ*_*i*_ an Ising variable and **e**_*i*_ one of the three possible anisotropy directions of the kagome lattice. To scale the temperature, we rely on the effective nearest-neighbour coupling *J*_nn_=*J*_1_/2+7*D*/4. This coupling quantifies the nearest-neighbour effective interaction between two nanomagnets, that is, the absolute temperature at which the model is expected to enter the first spin-ice manifold, SI1. Following our previous work^17^, *J*_1_ is chosen such that *J*_nn_=5*D*, to account for the multipolar terms mentioned above.

### *Ab initio* coding of emergence

**Figure 5:**
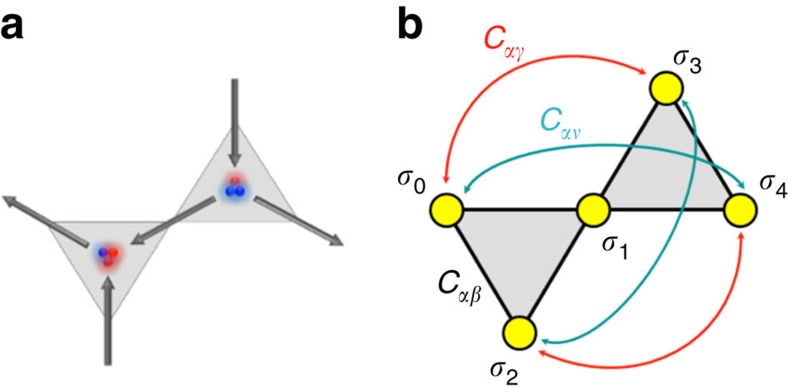
Definition of the magnetic charges at the vertices and of the spin–spin correlations. Every spin is the connection point between two vertex charges and contributes with a +1 magnetic charge in one triangle and −1 in the other. (**a**) A (*σ*_0_, *σ*_1_, *σ*_2_, *σ*_3_, *σ*_4_)=(−1, +1, +1, −1, +1) configuration for the kagome spin ice. (**b**) In the general case, the vertex charges can be found by summing up the spin scalar values per triangle (*σ*_*i*_). The constituting spins of a charge correlation pair form *αβ*, *αγ* and *αν* pairs.

Given the fractionalization of the spins into opposite magnetic poles, the total magnetic charge for each kagome vertex can be written as the sum of three individual charge contributions (see Fig. 5a). Using the scalar values (*σ*_*i*_=±1) that define the orientation of each lattice spin (**S**_**i**_) with respect to their local anisotropy axis (**e**_**i**_), that is, **S**_**i**_=*σ*_*i*_**e**_**i**_, the value of each vertex charge can be expressed as the sum of these local spin scalars,


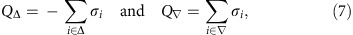


where *Q*_Δ_ and *Q*_∇_ are the values of the vertex charges of a Δ-shaped/∇-shaped kagome triangle, while the − sign ensures global charge neutrality. A nearest-neighbour charge–charge term, *Q*_*u*_·*Q*_*v*_, where *u* and *v* are the indices of the hexagonal lattice, involves the product of two *Q*_Δ,∇_ charges, which can be expanded into a summation of *σ*_*i*_·*σ*_*j*_ pairs. Furthermore, by conveniently arranging the resulting pairs into their corresponding correlation classes (Fig. 5b), this charge–charge term can be expressed as a linear combination of the first three pairwise spin correlators,


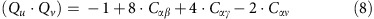


This relation between the nearest-neighbour charge correlator and the spin correlators is the cornerstone of the hybrid spin–charge model description, which can be tailored out of the dipolar Hamiltonian,


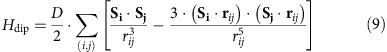


where **r**_**ij**_ stands for the relative position vector between the two spins **S**_**i**_ and **S**_**j**_, and *D* is the dipolar coupling constant. Note that the dipolar Hamiltonian involves all spin–spin correlations, in particular the ones needed in equation (8). Expanding the dipolar couplings and grouping them appropriately allows to rewrite the Hamiltonian as:


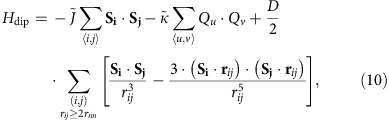


which will be written, for the sake of clarity,


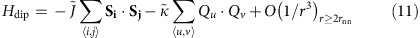


with 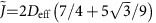, 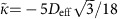, 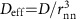 and *r*_nn_ the nearest-neighbour distance. Since the coupling constants 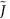 and 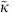 are positive and negative, respectively, they correspond to a ferromagnetic coupling between the nearest-neighbouring spins and to an antiferromagnetic coupling between the nearest-neighbouring vertex charges. As mentioned in the manuscript, the first one ensures that ice rules are fulfilled, while the second one leads to charge crystallization at lower temperatures, as 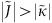. Note also that the charge–charge term is fully compatible with the spin–spin coupling and is not frustrated as it is defined on an hexagonal lattice. The remaining longer-range couplings play an important role as well, as they ultimately lead to the magnetic ordering at lowest temperatures. This magnetic ordering does not interfere with the first two terms of the hybrid spin–charge model: it is compatible with the kagome ice rules, the magnetic charge crystal, and the absolute convergence of the series of the longer-range couplings shows that this ordering takes place at temperatures lower than 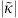 (and hence than 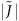 as well).

## Additional information

**How to cite this article:** Canals, B. *et al*. Fragmentation of magnetism in artificial kagome dipolar spin ice. *Nat. Commun.* 7:11446 doi: 10.1038/ncomms11446 (2016).

## Supplementary Material

Supplementary Information Supplementary Figures 1-2, Supplementary Table 1, Supplementary Notes 1-2 and Supplementary References
