# Supplementary material for: Fragmentation of magnetism in artificial kagome dipolar spin ice
Source: Nat Commun. 2016 May 13;7:11446. doi: 10.1038/ncomms11446 (PMC4869173; doi:10.1038/ncomms11446)
Supplement: Supplementary Information — Supplementary Figures 1-2, Supplementary Table 1, Supplementary Notes 1-2 and Supplementary References [file ncomms11446-s1.pdf]

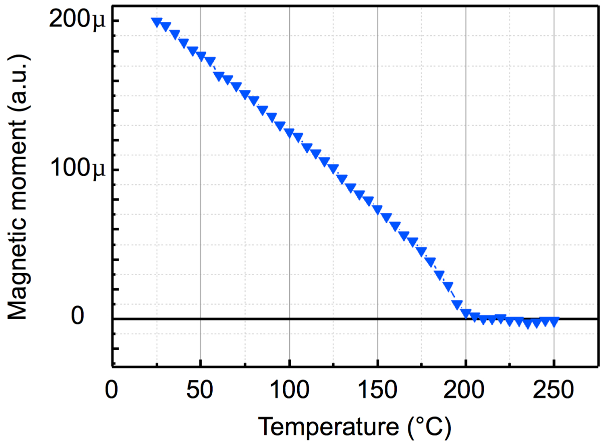

Supplementary Figure 1: **Temperature dependence of the GdCo thin film**. Temperature dependence of the magnetic moment of the 10 nm-thick GdCo film used to fabricate our thermally active, artificial kagome spin ice systems. Measurements are obtained using a vibrating sample magnetometer.

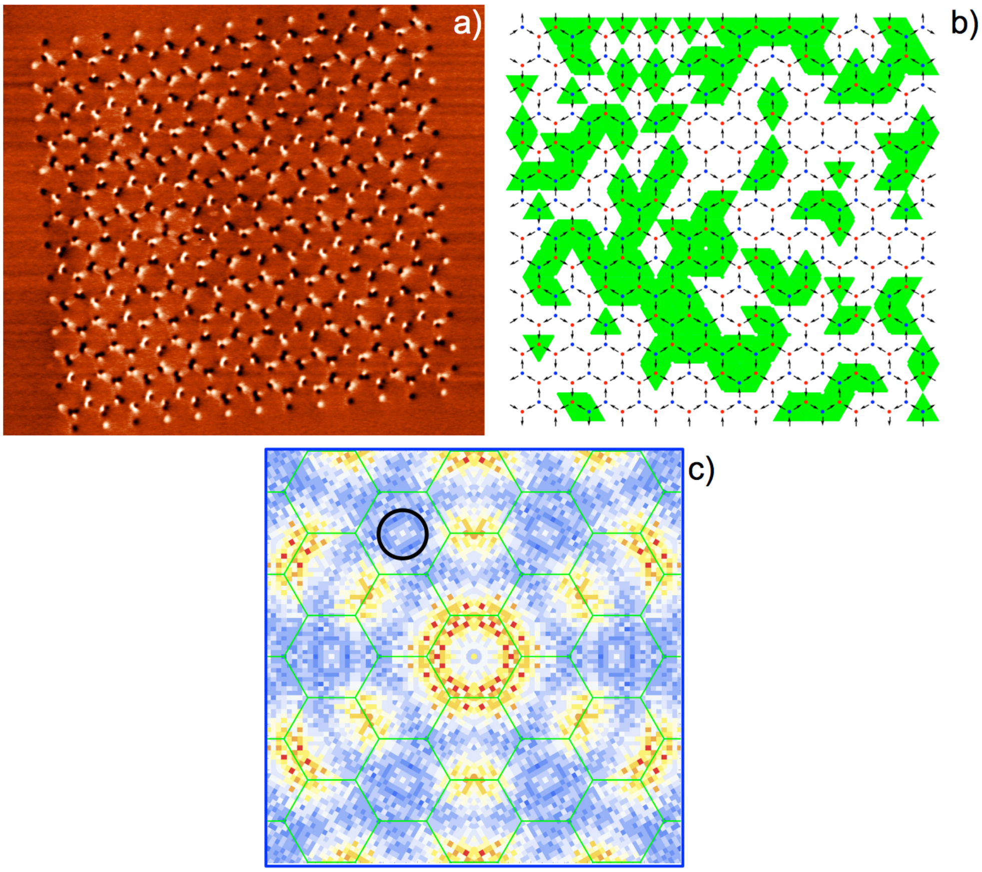

Supplementary Figure 2: **Experimental evidence of spin fragmentation in a field demagnetized athermal sample.** (a) 23x21 μm^2 magnetic force microscopy image of a 30 nm-thick permalloy kagome spin ice array. (b) Analysis of the magnetic image shown in (a). Arrows indicate the local direction of the Ising spins, red/blue dots represent the magnetic charge state at each vertex, and the white/green domains illustrate the corresponding magnetic charge crystallites. (c) Magnetic structure factor of the spin configuration shown in (b). In this 2D map, we observe Bragg peaks (see black circle) together with a structured background (regions in yellow), thus demonstrating the existence of a spin fragmentation process.

$C_{\alpha\beta}$	$C_{\alpha\gamma}$	$C_{\alpha\nu}$	$C_{\alpha\delta}$	$C_{\alpha\tau}$	$C_{\alpha\eta}$	$C_{\alpha\varphi}$	Q_iQ_{i+1}
0.167	−0.161	−0.010	−0.283	0.002	−0.004	0.024	−0.273

Supplementary Table 1: Spin-spin and charge-charge correlators deduced from the magnetic configuration shown in Supplementary Figure 2(a-b), which contains 552 spins. From the value of the nearest-neighbor spin correlator ($C_{\alpha\beta}$), we show that the kagome ice rule is satisfied everywhere in the lattice (no 3-in or 3-out forbidden state). The configuration is well demagnetized and clear signatures of longer-range dipolar interactions are visible, for example, through the low negative values of the $C_{\alpha\gamma}$ and $C_{\alpha\delta}$ spin correlations.

Supplementary Note 1: additional material properties

The magnetic properties of our GdCo thin films have been studied using conventional magnetometry. The spontaneous magnetisation M_s and the Curie temperature T_C of this ferrimagnetic alloy have then been determined. At room temperature, M_s is estimated to be 355 kA/m, and T_C = 200°C (see Supplementary Figure 1). These values are in agreement with the systematic study of P. Hansen et al. (see Supplementary Ref. [1]). In addition, the magnetization lies into the plane of the film and no in-plane anisotropy has been measured. We also have investigated the effect of annealing on the magnetic properties. A first annealing at 500 K results in a decrease of the coercivity without any change of the spontaneous magnetization. A subsequent identical annealing has no influence anymore.

Supplementary Note 2: additional experimental evidence of spin fragmentation

Our observation of a spin fragmentation process is not limited to the case of thermally active, GdCo kagome ice systems. Very similar results have been obtained with athermal, 30 nm-thick permalloy-based kagome arrays, that we demagnetized using a field protocol prior to imaging them with a Magnetic Force Microscope [see Supplementary Figure 2(a)]. In these types of samples, we also observed Bragg peaks in the 2D maps of the magnetic structure factor together with the diffuse background associated to the divergence-free channel [see Supplementary Figure 2(c)], thus proving the generality of the concept and the capability to measure it experimentally using different materials and demagnetization protocols. To demagnetize the array, the sample is rotated at ∼10 Hz in a damped, in-plane sinusoidal magnetic field (20 seconde period, ∼3 μT amplitude variation between two periods, from 60 to 0 mT, i.e. ∼110 hour demagnetization), and the overall protocol has been applied twice.

To demonstrate that the array is well-demagnetized, we provide below the values of different quantities determined from the image reported in Supplementary Figure 2(a) and analyzed in Supplementary Figure 2(b), particularly the nearest-neighbor charge correlator and the first 7 spin-spin correlators. These values (see Supplementary Table 1) are overall representative of those predicted by thermodynamics for an effective temperature of T/J_{nn}=0.063. Note that this effective temperature is very low for an athermal system that has been demagnetized using a field protocol. With this type of sample, most of the time the effective temperature is of the order to T/J_{nn}=1, even in cases where signatures of dipolar interactions are observed [2]. In fact, this effective temperature is comparable to the one deduced from our measurements on thermally active GdCo system (T/J_{nn}=0.051). This shows that long (110 hours) demagnetization protocol can be efficient as well to bring kagome lattices in their low-energy manifold, where exotic physics emerges.

Supplementary References

[1] Hansen, P. et al. Magnetic and magneto-optical properties of rare-earth transition-metal alloys containing Gd, Tb, Fe, Co. *J. Appl. Phys.* **66**, 756-767 (1989).

[2] Rougemaille, N. et al. Artificial kagome arrays of nanomagnets: a frozen dipolar spin ice. *Phys. Rev. Lett.* **106**, 057209 (2011).
